# Auditory steady state responses elicited by silent gaps embedded within a broadband noise

**DOI:** 10.1186/s12868-022-00712-0

**Published:** 2022-05-06

**Authors:** Seiichi Kadowaki, Takashi Morimoto, Hidehiko Okamoto

**Affiliations:** 1 Department of Physiology, International University of Health and Welfare Faculty of Medicine Graduate School of Medicine, 4-3 Kozunomori, Narita, 286-8686 Japan; 2 Department of Audiological Engineering, RION Co., Ltd., Tokyo, 185-8533 Japan

**Keywords:** Auditory steady state response (ASSR), Electroencephalography (EEG), Gap, Human, Speech perception, Temporal processing

## Abstract

**Background:**

Auditory temporal processing plays an important role in speech comprehension. Usually, behavioral tests that require subjects to detect silent gaps embedded within a continuous sound are used to assess the ability of auditory temporal processing in humans. To evaluate auditory temporal processing objectively, the present study aimed to measure the auditory steady state responses (ASSRs) elicited by silent gaps of different lengths embedded within a broadband noise. We presented a broadband noise with 40-Hz silent gaps of 3.125, 6.25, and 12.5 ms.

**Results:**

The 40-Hz silent gaps of 3.125, 6.25, and 12.5 ms elicited clear ASSRs. Longer silent gaps elicited larger ASSR amplitudes and ASSR phases significantly differed between conditions.

**Conclusion:**

The 40 Hz gap-evoked ASSR contributes to our understanding of the neural mechanisms underlying auditory temporal processing and may lead to the development of objective measures of auditory temporal acuity in humans.

**Supplementary Information:**

The online version contains supplementary material available at 10.1186/s12868-022-00712-0.

## Background

Hearing ability is typically assessed using pure tone audiometry; however, pure tone thresholds alone do not explain a person's auditory skills. We need various auditory skills for speech comprehension, especially auditory temporal processing plays a major role in speech recognition [1]. Auditory temporal resolution implies the ability to extract temporal envelopes of sound signals, and is considered to be essential, particularly in discriminating each consonant [2].

Several behavioral measures have been developed to evaluate auditory temporal resolution in humans. One of these is a gap detection behavioral test in which a subject listens to a series of sounds with a short silent interval, called “gap”, and reports whether they perceived it or not [3, 4].

The gap detection thresholds (GDT) depended on the test sound types [5]. When pure tones were used as test sound signals, the GDT was 2–3 ms when the same test tone was used before and after the silent gap (within-frequency-channel processing), and GDT was larger when the test tone frequencies differed before and after the silent gap (across-frequency-channel processing) [6, 7]. One major problem in measuring GDT using pure tones is that spectral splatter is generated by the onset and offset of the tone signals. Test subjects might detect this spectral splatter rather than the temporal gap itself. To eliminate this issue, white noise is often used as a test sound signal since the gap embedded within a white noise does not generate the spectral splatter. Previous studies reported that GDT for gaps embedded within a white noise ranged between 2 and 3 ms in normal hearing individuals [8]; however, people with poor speech perception showed longer GDT [9–11]. Nair et al. [12] demonstrated that the word discrimination ability was negatively correlated with the GDT as measured in old people with normal hearing.

Since the gap detection test requires a subject to respond to the presence or absence of a gap signal, it is difficult for infants, children, and people with cognitive impairments to perform the test. To obtain hearing thresholds without active responses by subjects, auditory evoked brain responses are often recorded using electroencephalography (EEG). The auditory brainstem response (ABR) and auditory steady-state response (ASSR) are often used in clinical settings. ABR is an auditory evoked response that is generally recorded within 10 ms of the application of a click stimulus. ABR is a standard measure to evaluate hearing thresholds in clinical practice and represents neural activity in the periphery to the brainstem of the auditory pathway [13]. ASSR is another auditory evoked response that is used for objective hearing threshold measurements. It is a sinusoidal steady-state auditory evoked response elicited by sounds with a periodic temporal structure. The most prominent ASSR could be obtained at a modulation frequency of 40 Hz [14] and the generator of the 40 Hz ASSR appears to be the primary auditory cortex [15, 16] which plays a major role in the neural processing of gap signals [17, 18].

ABR and ASSR enable objective measurements of hearing thresholds; however, a standard objective measure of auditory temporal resolution has not yet been available Previous studies demonstrated that temporal gaps embedded within a continuous sound could elicit mismatch negativity (MMN) and N1-P2 responses and the amplitudes of MMN and N1-P2 responses were significantly correlated with the lengths of silent gaps [19–21]. However, to the best of our knowledge, there is no study that investigated the effects of gap lengths on ASSR.

Therefore, the goal of the present study was to measure ASSR elicited by 40-Hz silent gaps of different lengths embedded within a broadband noise using EEG and reveal the effects of gap lengths on the ASSR amplitudes and phases.

## Methods

### Participants

Twenty students (7 males) with normal pure-tone audiometry were recruited at the International University of Health and Welfare for this experiment. Their ages ranged between 19 and 30 years (median 20 years). None of the participants had neurological or psychiatric disorder and took any drugs, including alcohol, within 24 h prior to the experiment. They were fully informed about the study and gave written informed consent for their participation. The present study was approved by the Ethics Committee of the International University of Health and Welfare, School of Medicine and conformed to The Code of the World Medical Association (Declaration of Helsinki).

### Behavioral gap detection threshold (GDT)

GDT was measured in all participants. An adaptive, 3-alternative forced-choice, 2-down 1-up procedure was used to track the 70.7% correct rate for GDT determination as described in detail in the previous studies [22, 23]. The length of the white noise (sampling rate: 48,000 Hz) was set to 500 ms; the silent gap was inserted in the center. The inter-stimulus interval between two successive test sounds was 500 ms. The silent gap length started from 7 ms. The step size was set to 1 ms in the first four reversals and 0.5 ms thereafter. The measurements were continued for 12 reversals, and the threshold was estimated as the mean of the values for the last eight reversals. Thresholds were measured twice, and the mean of the two measurements was used as the GDT.

### Stimuli and experimental design for EEG recording

**Fig. 1:**
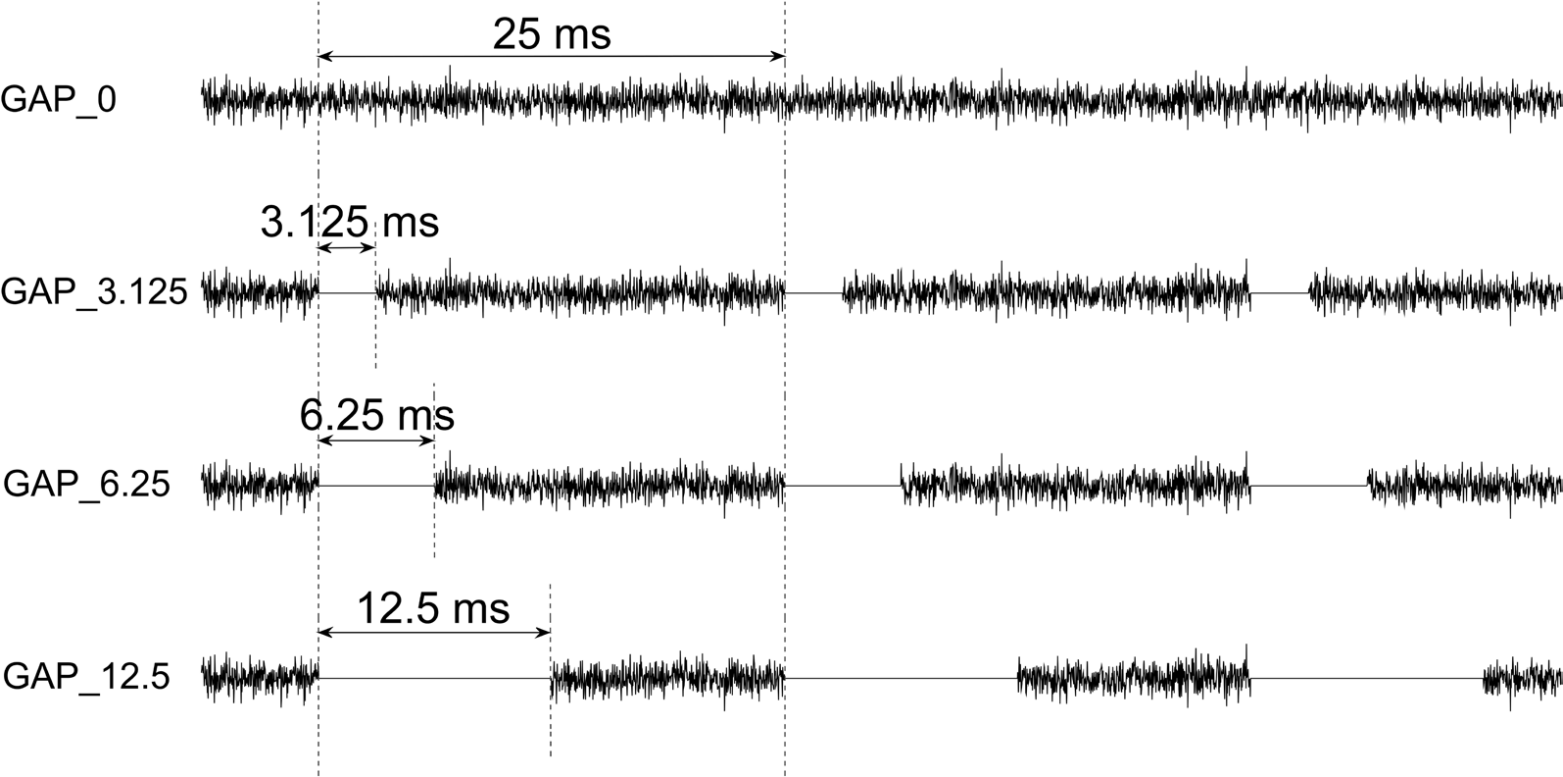
Schematic display of the auditory stimulation. Examples of test sound stimuli with silent gaps of 0 (GAP_0), 3.125 (GAP_3.125), 6.25 (GAP_6.25), and 12.5 ms (GAP_12.5) are depicted from top to bottom, respectively. For sample audio files, access Online Resource 1.

We prepared 20 white noises (sampling rate: 48,000 Hz) having the same intensity with a duration of one minute as test sound stimuli for EEG measurements by using MATLAB R2020a (The MathWorks Inc., MA, USA). Then, we divided them into four types of test sound stimuli. One type was a continuous white noise with no silent interval (GAP_0), and the others were white noises with silent gaps with durations of 3.125 ms (GAP_3.125), 6.25 ms (GAP_6.25), and 12.5 ms (GAP_12.5) (Fig. 1). Silence was inserted every 25 ms to match the modulation frequency of 40 Hz. More specifically, 21.875, 18.75, and 12.5 ms white noises were followed by 3.125, 6.25, and 12.5 ms silent gaps in GAP_3.125, GAP_6.25, and GAP_12.5 conditions, respectively. These gap lengths were exponentially equally spaced and were determined based on GDT and the modulation period (25 ms). We adopted half of the modulation period (12.5 ms) as the maximum gap length and one-eighth of the modulation period (3.125 ms) as the minimum gap length that is slightly larger than the GDT in normal hearing individuals (2–3 ms) [8]. The exemplary sound files are given in Additional File 1: GAP_0, GAP_3.125, GAP_6.25, and GAP_12.5 were randomly played for 20 min, resulting in five minutes in total for each gap condition.

Participants were presented with GAP_0 at an intensity of 70 dBA SPL via ER-3A insert earphones (Etymotic Research Inc., IL, USA). EEG recordings were performed with participants seated comfortably in a silent electromagnetically shielded room. They were instructed to watch a silent movie with captions during experiments.

### Data acquisition and analysis

Sound stimuli were presented via Multi Trigger System Ver.2 (MTS0410, Medical Try System, Co., Ltd., Japan), which simultaneously sent electric triggers to Neurofax EEG1200 (Nihon Koden, Co., Ltd., Japan). We used four types of triggers and each trigger was synchronized with the onset of each sound stimulus (GAP_0, GAP_3.125, GAP_6.25, or GAP_12.5). The EEG signals were recorded using a Neurofax EEG1200 system at a sampling rate of 1000 Hz. The recording electrodes (Ag/AgCl) were located at Cz, Fz, C3, and C4 according to the international 10–20 system. The data from Fz, C3, and C4 were used to reduce the electro-cardiac signals in Cz by ECG elimination filter implemented in the Neurofax EEG1200 system. Therefore, the data from Cz were used for the statistical analysis. The average signal of two electrodes placed on both mastoids was used as a reference, and the ground electrode was located at Fpz around the forehead midpoint. Electrode impedance was maintained below 15 kΩ. Recorded EEG data were exported as ascii files and were analyzed offline using Matlab R2020a and EEGLAB [24].

For EEG waveforms at Cz, a fast Fourier transform was computed in each condition and amplitude spectra were extracted after removing the powerline fluctuations at 50 Hz using the Clean-Line plugin for EEGLAB. In order to obtain ASSR, an epoching procedure was applied to the EEG signals. Using Matlab, markers were inserted into the EEG data at the time points of gap onsets (the end of broadband noise) and gap offsets (the beginning of broadband noise) of the gap-embedded sound stimulus (GAP_3.125, GAP_6.25, or GAP_12.5) based on the trigger synchronized with the sound stimulus onset. One test sound contained 2400 silent gaps and five test sounds were presented for each GAP condition. In total, 12,000 gap onset markers and 12,000 gap offset markers were labeled for each GAP condition of each participant. EEG waveforms at Cz were bandpass filtered (32–48 Hz) offline and epochs of 0 to 24 ms (25 sampling points) from the markers were averaged after artifact rejection (set to a threshold of ± 50 μV) separately for each gap-embedded noise condition (GAP_3.125, GAP_6.25, or GAP_12.5) for each participant. The obtained ASSR waveforms were fitted into the 40 Hz sinusoidal curves Y = a* sin(X–b), then the amplitudes (a) and the phase delays (b) were used for the statistical analysis.

Pearson correlation coefficients were calculated to assess the linear relationships between GDT and ASSR amplitudes for GAP_3.125, GAP_6.25, and GAP_12.5 conditions. ASSR amplitudes were evaluated using a repeated-measures analysis of variance (ANOVA) using GAP (GAP_3.125, GAP_6.25, and GAP_12.5) as a factor. The ASSR phase delay was calculated from the gap onset (the end of broadband noise) and from the gap offset (the beginning of broadband noise), respectively. ASSR phase delays from the gap onset and gap offset were evaluated using one-way repeated-measures ANOVA using GAP (GAP_3.125, GAP_6.25, and GAP_12.5) as a factor, respectively. For all the ANOVA tests, sphericity was assessed for each within-subject factor with Mauchly’s Test [25] and the degrees of freedom were corrected using Greenhouse–Geisser epsilon when appropriate. Bonferroni corrected paired *t*-tests were performed for post hoc multiple comparisons of GAP factors. All statistics were conducted using IBM SPSS Statistics Version 21 for Windows (Armonk, NY: IBM Corp.) and significance was accepted at *p* < 0.05.

## Results

**Fig. 2:**
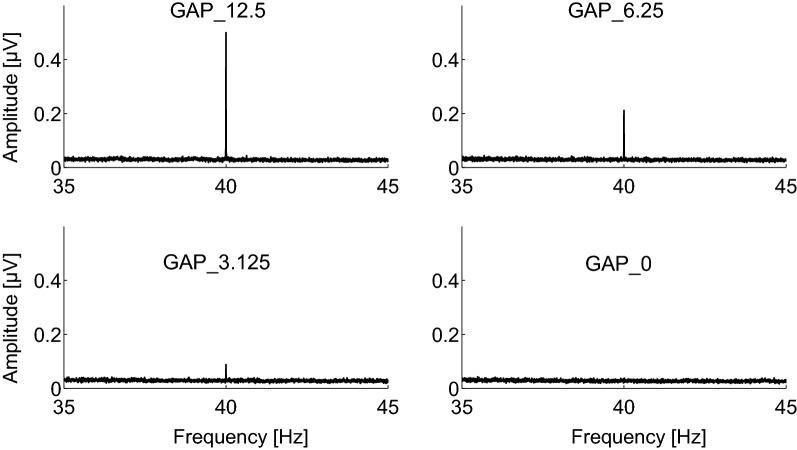
Group means (N = 20) of the EEG amplitude spectra. Grand averaged (N = 20) EEG amplitude spectra corresponding to GAP_12.5 (top left), GAP_6.25 (top right), GAP_3.125 (bottom left), and GAP_0 (bottom right) at Cz.

All participants completed GDT measurements. The mean and standard deviation of their GDT were 2.56 and 0.27 ms. The mean rejection rate of artifact-contaminated EEG epochs was 6.3%. Figure 2 shows the fast Fourier transformation waveforms averaged across all the participants under each condition. Figure 2 shows no prominent peak at 40 Hz in the continuous white noise condition (bottom right), whereas prominent 40 Hz peaks are visible in all the GAP conditions. The longer GAP conditions induced the larger 40 Hz power spectral peaks.

**Fig. 3:**
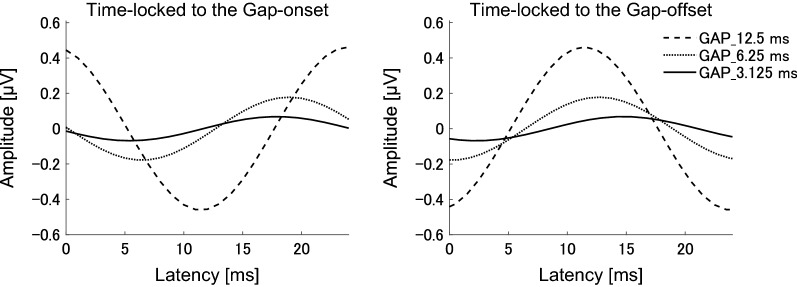
Grand averaged (N = 20) auditory steady state responses (ASSRs) elicited by 40-Hz silent gaps. The graph displays the grand-averaged waveforms of participants (N = 20). The dashed, dotted, and solid lines represent GAP_12.5, GAP_6.25, and GAP_3.125 conditions, respectively (see legends in the right upper corner).

**Fig. 4:**
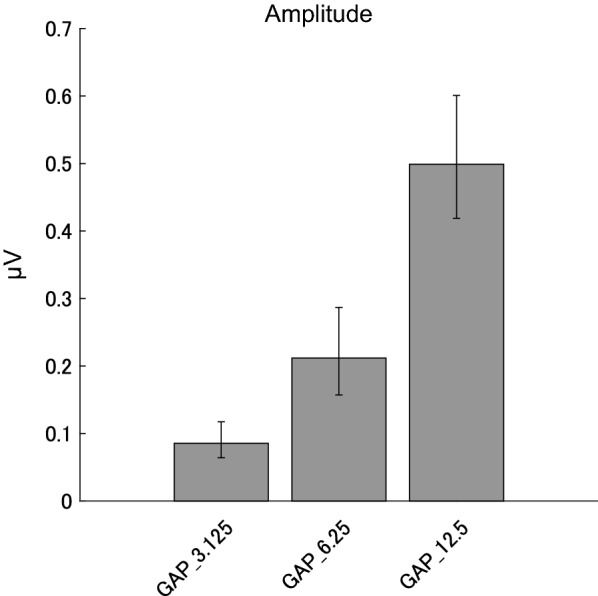
Group means (N = 20) of ASSR amplitudes. Group means (N = 20) of ASSR amplitudes elicited by silent gaps of 0, 3.125, 6.25, and 12.5 ms embedded within broadband noises at Cz. The error bars denote 95% confidence intervals.

Figure 3 demonstrates grand averaged EEG waveforms under GAP_3.125, GAP_6.25, and GAP_12.5 conditions. The waveforms represented clear 40 Hz sinusoidal curves. Figure 4 shows the mean ASSR amplitudes under each GAP condition together with the corresponding 95% confidence intervals obtained by boot-strap resampling tests (iteration = 100 000). There was no significant correlation between the GDT and the ASSR amplitudes in GAP_3.125 (r (18) = 0.287, *p* = 0.219), between the GDT and the ASSR amplitudes in GAP_6.25 (r (18) = 0.276, *p* = 0.239), and between the GDT and the ASSR amplitudes in GAP_12.5 (r (18) = 0.127, *p* = 0.595).

The one-way repeated-measures ANOVA applied to the ASSR amplitude revealed a significant main effect for GAP (F (1.162, 22.072) = 111.124, *p* < 0.00001). Post hoc multiple comparisons revealed significant differences between GAP_3.125 and GAP_6.25 (t (19) = − 5.706, *p* < 0.0001), GAP_3.125 and GAP_12.5 (t(19) = − 10.698, *p* < 0.00001), and GAP_6.25 and GAP_12.5 (t (19) = − 13.683, *p* < 0.00001). The ASSR amplitude significantly increased with an increase in the gap length.

**Fig. 5:**
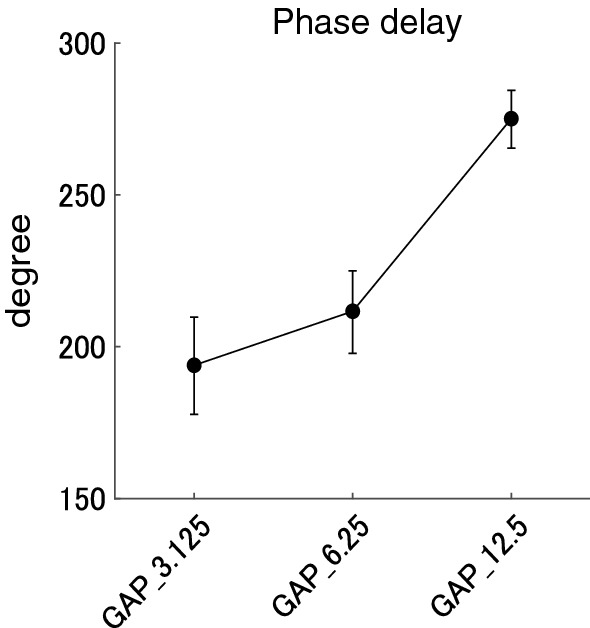
Group means (N = 20) of the ASSR phase delays from the gap onset. Group means of the ASSR phase delays from the gap onset (offset of the continuous noise) elicited by silent gaps of 3.125, 6.25, and 12.5 ms embedded within broadband noises at Cz. Error bars denote 95% confidence intervals.

Figure 5 shows mean phase delays from the gap onset in each GAP condition together with the corresponding 95% confidence intervals obtained by boot-strap resampling tests (iteration = 100 000). The phase delays in GAP_3.125, GAP_6.25, and GAP_12.5 ranged between 131.7 and 260.1 degrees (mean 193.8 degree), 149.4 and 260.2 degrees (mean 211.6 degree), 233.8 and 307.7 degrees (mean 275.1 degree), respectively. The one-way repeated-measures ANOVA applied to the ASSR phase delays from the gap onset resulted in a significant main effect for GAP (F (1.485, 28.223) = 99.185, *p* < 0.00001). Post hoc multiple comparisons revealed significant differences, GAP_3.125 and GAP_12.5 (t (19) = − 12.001, *p* < 0.00001), and GAP_6.25 and GAP_12.5 (t (19) = − 16.272, *p* < 0.00001), but no significant difference between GAP_3.125 and GAP_6.25 (t (19) = − 2.526, *p* = 0.062). The ASSR phase delayed with an increase in the gap length.

**Fig. 6:**
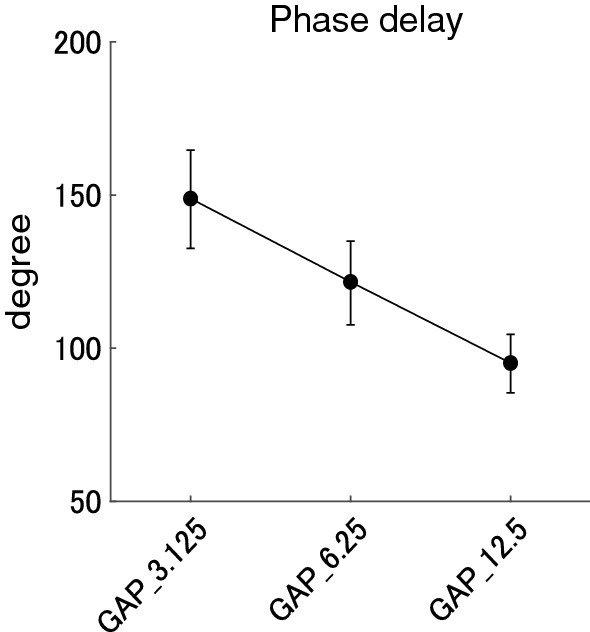
Group means (N = 20) of the ASSR phase delays from the gap offset. Group means of the ASSR phase delays from the gap offset (onset of the continuous noise) elicited by silent gaps of 3.125, 6.25, and 12.5 ms embedded within broadband noises at Cz. Error bars denote 95% confidence intervals.

Figure 6 shows mean phase delays from the gap offset in each GAP condition together with the corresponding 95% confidence intervals obtained by boot-strap resampling tests (iteration = 100 000). The phase delays in GAP_3.125, GAP_6.25, and GAP_12.5 ranged between 86.8 and 215.1 degrees (mean 148.8 degree), 59.4 and 170.2 degrees (mean 121.6 degree), 53.8 and 127.7 degrees (mean 95.1 degree), respectively. The one-way repeated-measures ANOVA applied to the ASSR phase delays from the gap offset resulted in a significant main effect for GAP (F (1.485, 28.223) = 39.166, *p* < 0.00001). The post hoc multiple comparisons revealed significant differences between GAP_3.125 and GAP_6.25 (t (19) = 3.879, *p* < 0.004), GAP_3.125 and GAP_12.5 (t (19) = 7.930, *p* < 0.00001), and GAP_6.25 and GAP_12.5 (t (19) = 6.776, *p* < 0.00001). Contrary to the phase delays from the gap onset (Fig. 5), the ASSR phase gradually advanced with an increase in the gap length.

## Discussion

The results of the present study demonstrated that clear ASSRs were elicited by 40-Hz silent gaps embedded within a broadband noise and the amplitude and phase of the gap-evoked ASSR significantly depended on the gap length. Usually, ASSR is elicited by the appearance of the sound signals and sound types significantly influenced the amplitude and phase of 40-Hz ASSR [14]. In the present study, we used the disappearance of sound signals as test stimuli and successfully obtained prominent 40-Hz ASSR elicited by silent gaps of 3.125, 6.25, and 12.5 ms (see Figs. 2 and 3). Moreover, the ASSR amplitude increased in proportion to the length of the silent gaps as shown in Fig. 4. The phase delays of gap-evoked ASSR significantly differed between GAP conditions (GAP_3.125, GAP_6.25, and GAP 12.5, see Figs. 5 and 6). When the phase delays were calculated from the gap onset (or the offset of the noise segment) phases were delayed as the gap became longer (Fig. 5). In contrast, when the phase delays were calculated from the gap offset (or the onset of the noise segment) they advanced as the gap became longer (Fig. 6). A previous study demonstrated that the phase of ASSR advanced and the amplitude increased as the intensity of the stimuli increased from 35 to 75 dB SPL [26]. Furthermore, the N1 response elicited by a longer gap had a shorter latency and larger amplitude [27]. These findings support the hypothesis that not the offset, but the onset of broadband noise (or the offset of the gap) would play a major role in triggering ASSR.

Previous studies reported that the gaps in continuous sounds could elicit the auditory evoked responses, such as MMN, N1, and P2. The amplitude of MMN has been shown to increase in proportion to the gap length [21]. Moreover, the study demonstrated more delayed and smaller amplitude MMN responses to silent gaps in older adults with normal hearing than in younger adults despite the absence of behavioral differences in gap detection. A previous study showed that N1 and P2 responses in healthy adults were obtained when the gap length was 5 ms or longer and also that the amplitudes of N1 and P2 were smaller when the gap length became shorter [28]. The present study also showed that the longer gap embedded within a continuous sound elicited the larger and more advanced ASSR time-locked to the gap offset than the shorter gap. ASSR has different characteristics with other auditory evoked responses, such as N1, P2, and MMN. ASSR is less sensitive to alertness and attention than slow auditory evoked responses and MMN. Previous studies demonstrated that auditory attention enhanced ASSR [29, 30], whereas others did not [31–34]. Even though attention may modulate the ASSR amplitude, the attentional effect on ASSR appears to be smaller than that on slow auditory evoked responses [35]. Furthermore, ASSR is more stable against aging than other auditory evoked responses. MMN was significantly smaller in healthy elderly individuals than in younger adults [21, 36, 37] and significant age-related changes have been reported in N1, P2, N2, and P300 [38, 39]. In contrast, normal aging appears to have less influence on the amplitude of 40-Hz ASSR [40, 41]. A previous study compared the amplitude of 40-Hz ASSR in young adults and elderly individuals and found no significant differences in the amplitude or phase of 40-Hz ASSR between these age groups [41]. These previous studies suggest that the gap-evoked ASSR may represent neural activity more specific to the auditory temporal processing than other slow auditory evoked components.

The neural processing of silent gaps appears to differ from that of normal sound stimuli. Previous studies reported that lesions of auditory cortex did not affect the detection of sound increments (such as noise bursts) but deteriorated the detection of brief sound decrements (such as gaps in noise) [17, 18, 42–44]. A previous study reported that the N1 response elicited by gaps in a continuous noise had distinct scalp distribution and intracranial neural sources compared to that elicited by clicks [27]. Another study demonstrated that the ASSR amplitudes elicited by the brief tones increased as stimulus duration decreased [45], whereas our results showed that the ASSR amplitudes elicited by the silent gaps decreased as the gap duration decreased. These findings suggested that the ASSR elicited by the gaps of different lengths in the present study at least partially differed from those elicited by presentations of sound signals such as tone bursts, amplitude-modulated tones, noises and clicks.

Regarding the clinical importance of measuring the temporal resolution in humans, previous studies found that the gap detection test was effective in identifying auditory processing disorder [46], children with reading and writing difficulties [47], autism [48], and even Alzheimer's disease [49]. However, since temporal resolution is usually assessed by means of behavioral tasks, there is a serious concern that the deteriorations in thresholds that appeared after monotonous behavioral testing in those people who cannot reliably complete behavioral tests may be caused by psychological or psychiatric factors other than auditory processing disorder. ASSR is a stable and completely objective test that does not require the participant's active responses. Therefore, the gap-evoked ASSR obtained in the present study appears to be especially beneficial to non-invasively and objectively assess the auditory temporal processing in the above disorders. However, the limitation of this experiment is that only people with normal hearing and GDT participated in the present study. It remains unsolved whether gap-evoked ASSRs measured in people with deteriorated auditory temporal processing differ from those obtained in the recent study. Thus, it is necessary to measure the gap-evoked ASSR in people with prolonged GDT in the future studies.

## Conclusions

The present results showed that 40-Hz silent gaps embedded within broadband noise elicited significant ASSR. The ASSR amplitude increased and the ASSR phase from the gap offset advanced in proportion to the gap length. The gap-evoked ASSR would contribute to our understanding of the neural mechanisms underlying auditory temporal processing and be applied to objectively measure auditory temporal acuity in humans.

## Supplementary Information

**Additional file 1.** Samples of test sound stimuli. The file contains examples of GAP sound stimuli used in the present study (sampling rate: 48,000 Hz). GAP_0 is a white noise. GAP_3.125, GAP_6.25, and GAP_12.5 contain silent gaps with durations of 3.125, 6.25, and 12.5 ms at a rate of 40 Hz, respectively. Sound waveforms are displayed in Fig. 1.

## Data Availability

The datasets used and/or analyzed during the present study are available from the corresponding author on reasonable request.

## Abbreviations

ABR: Auditory brainstem response
ANOVA: Analysis of variance
ASSR: Auditory steady state response
EEG: Electroencephalography
GDT: Gap detection threshold
MMN: Mismatch negativity
